# Vascular and Myocardial Structure and Function in Adolescents with Type 1 Diabetes: The CARDEA Study

**DOI:** 10.1155/2023/8662038

**Published:** 2023-08-24

**Authors:** Soren Harnois-Leblanc, Vanessa McNealis, Matthias G. Friedrich, Jean-Luc Bigras, Andraea Van Hulst, Anne Monique Nuyt, Tracie A. Barnett, Andrea Benedetti, Marie-Ève Mathieu, Vicky Drapeau, Marie-Pierre Sylvestre, Mélanie Henderson

**Affiliations:** ^1^Research Center of the Sainte-Justine University Hospital, Montréal, QC, Canada; ^2^Department of Preventive and Social Medicine, School of Public Health, Université de Montréal, Montréal, QC, Canada; ^3^Research Center of the Montréal Hospital University Center, Montréal, QC, Canada; ^4^Department of Epidemiology and Biostatistics, Faculty of Medicine, McGill University, Montréal, QC, Canada; ^5^McGill University Health Centre, Montréal, QC, Canada; ^6^Department of Medicine and Diagnostic Radiology, Faculty of Medicine, McGill University, Montréal, QC, Canada; ^7^Department of Pediatrics, Faculty of Medicine, Université de Montréal, Montréal, QC, Canada; ^8^Ingram School of Nursing, McGill University, Montréal, QC, Canada; ^9^Department of Family Medicine, Faculty of Medicine, McGill University, Montréal, QC, Canada; ^10^School of Kinesiology and Physical Activity Sciences, Faculty of Medicine, Université de Montréal, Montréal, QC, Canada; ^11^Department of Physical Education, Faculty of Education Sciences, Université Laval, Québec City, QC, Canada

## Abstract

**Introduction:**

Despite heightened risk of cardiovascular disease (CVD) among individuals with type 1 diabetes, few studies in this population have investigated the development of CVD using early markers in adolescence. We compared risk factors (blood pressure (BP) and dyslipidemia) and early markers of CVD between adolescents with and without type 1 diabetes and explored effect modification by sex.

**Methods:**

Cross-sectional study using data from the CARdiovascular Disease risk in pEdiatric type 1 diAbetes (CARDEA) study. We recruited 100 adolescents with type 1 diabetes at the Sainte-Justine University Hospital Center and 97 adolescents without diabetes (14–18 years). We measured arterial stiffness by carotid-femoral pulse wave velocity, endothelial function by brachial artery flow-mediated dilation test, as well as left ventricular (LV) mass, papillary mass, and wall thickness by cardiac MRI. We used multivariable linear regression models to assess the impact of type 1 diabetes on each outcome adjusting for age, sex, ethnicity, adiposity, and familial income.

**Results:**

Adolescents with type 1 diabetes had 0.21 standard deviations (SD) (95% CI: 0.04; 0.38) higher diastolic blood pressure *z*-score (zDBP), 0.21 mmol/L (95% CI: 0.02; 0.40) higher low-density lipoprotein cholesterol (LDL-c) levels, and 17% (95% CI: 4; 29) higher triglyceride levels and lower endothelial function based on acceleration (−77.4 cm/s^2^, 95% CI: −133.1; −21.6) compared with adolescents without diabetes. Girls with type 1 diabetes had higher systolic blood pressure *z*-score (zSBP), and boys with type 1 diabetes had lower LV mass and wall thickness compared to healthy peers.

**Conclusions:**

In addition to higher BP and abnormal lipid profiles, adolescents with type 1 diabetes present endothelial dysfunction and alterations in cardiac structure (in boys) compared to adolescents without diabetes, suggesting that CVD prevention should be incorporated into type 1 diabetes management early in the disease.

## 1. Introduction

Cardiovascular disease (CVD) mortality is sevenfold higher in adults with youth-onset type 1 diabetes compared to individuals without diabetes [1]. A high prevalence of CVD risk factors is documented in adolescents and young adults with childhood-onset type 1 diabetes [2]. State-of-the art methods based on vascular Doppler and cardiac magnetic resonance (CMR) imaging have gained interest to identify the earliest signs of CVD development in adolescents with type 1 diabetes [3, 4]. In particular, CMR is a useful method to measure early markers of CVD given the safety in children (no radiation), its excellent accuracy for quantitative data, three-dimensional (3D) capabilities, and high spatial resolution. The identification of alterations in vascular and myocardial structure and function is particularly relevant to inform on the pathophysiology of early CVD and potential targets for prevention among young people at-risk.

Arterial stiffness presents with impaired vessel distensibility and is usually assessed by pulse wave velocity (PWV) [5]. In adults with type 1 diabetes, greater arterial stiffness is predictive of end-stage kidney disease, cardiovascular events, and mortality [6]. Adolescents with type 1 diabetes show higher arterial stiffness (assessed by truncal PWV) compared to adolescents without diabetes (5.4 vs. 5.1 m/s, adjusted for age) [4]. Endothelial function, another early marker of CVD, is evaluated by the dilation of vessels in response to stimuli-induced nitric oxide release [7]. Adolescents and adults with type 1 diabetes have endothelial dysfunction compared to peers without diabetes [4, 8]. In addition to markers of vascular function, early signs of CVD can be assessed by investigating myocardial structure and function. Early myocardial alterations in function and structure that predate systolic and diastolic dysfunction in adults [9] can be detected by CMR. However, CMR data are very sparse in adolescents with type 1 diabetes, with the few available studies conducted in small samples. Heier et al. [10] (*n*_T1D_ < 50) and Schäfer et al [3] (*n*_T1D_ < 20) found no differences between adolescents with and without type 1 diabetes regarding left ventricular (LV) mass and ejection fraction. In the Schäfer et al.'s study [3], adolescents with type 1 diabetes exhibited LV discoordination, but no differences in traditional markers of LV circumferential strain.

Overall, studies reporting vascular and myocardial structure and function alterations in young people with type 1 diabetes remain scarce. In addition to having limited power, many of these studies did not address confounding. Notably, many studies failed to account for adiposity and socioeconomic status, despite their potential to confound the association between type 1 diabetes status and CVD risk in adolescents [11–15]. Evidence examining differences in CVD markers across adolescents with and without type 1 diabetes needs to account for these confounders going forward.

Sex differences in CVD risk are evident in adults with type 1 diabetes [16], and emerging evidence suggest that differences may already exist in adolescence. In particular, boys are more likely to present higher PWV and lower brachial artery distensibility [4, 14] compared to girls of the same age. Investigating sex differences in the early development of CVD among adolescents with type 1 diabetes will contribute to a better understanding of mechanisms implicated and the development of personalized preventive strategies.

Our study aims to compare established CVD risk factors (blood pressure and dyslipidemia) and markers of vascular and myocardial structure and function in a large sample of adolescents with and without type 1 diabetes accounting for confounders. A secondary exploratory objective is to test if sex is an effect modifier in the relationship between having type 1 diabetes and CVD risk.

## 2. Materials and Methods

### 2.1. Design and Participants

The present study is cross-sectional and its full protocol (the CARdiovascular Disease risk in pEdiatric type 1 diAbetes (CARDEA) study) is available elsewhere [17]. The main goal of the CARDEA study is to gain a better understanding of CVD development in adolescents with type 1 diabetes using novel imaging technology and to investigate its determinants. Recruitment took place between 2016 and 2019. Adolescents with type 1 diabetes aged between 14 and 18 years were recruited from the diabetes clinic at the Sainte-Justine University Hospital Center (Montreal, QC, Canada). Adolescents without diabetes were recruited through friends of participants with type 1 diabetes or in high schools, community sport clubs, and summer camps in the Greater Montreal area. Adolescents without diabetes were matched for age (±6 months) and sex based on frequency matching. Adolescents who have known pathologies influencing CVD risk (e.g., congenital cardiomyopathy), or had a major condition limiting the participation to the research evaluations, were deemed ineligible. For adolescents aged less than 18 years, written assent and parental consent were obtained; adolescents aged 18 years provided written consent. Ethical approval was obtained from the Sainte-Justine University Hospital Research Ethics Board (2016-936).

### 2.2. Data Collection

Research assessments were identical for participants with and without type 1 diabetes, except that participants with diabetes also attended their regular medical follow-up appointment at the diabetes clinic. Participants were welcomed in the morning at the Sainte-Justine University Hospital Clinical Research Unit following a 12 hr overnight fast. They were also asked to refrain from performing intense exercise during the 48 hr preceding the research evaluation. Fasting blood samples were obtained by venipuncture at 8:30 am. All biochemical analyses were conducted at the Sainte-Justine University Hospital Central Laboratory. Glycated hemoglobin (HbA1c) was measured from whole blood by high-performance liquid chromatography on a Tosoh G8 device (Tosoh Bioscience, San Francisco, USA). To obtain a representative measure of usual glycemic control in type 1 diabetes, we averaged the HbA1c value obtained at the research visit and the values retrieved from participants' medical health records during the year preceding the research visit (all measured using the same device).

### 2.3. Measurement of CVD Risk Factors

Total cholesterol, triglycerides, and high-density lipoprotein cholesterol (HDL-c) levels were measured from fasting whole blood samples by enzymatic methods on the Architect C8000/ci8200 (Abbott, Chicago, USA) automat using commercial colorimetric kits (Roche Diagnostic, Indianapolis, USA). Low-density lipoprotein cholesterol (LDL-c) levels were calculated with the Friedewald equation [18]. Systolic and diastolic blood pressure were measured on the right arm five times at 1 min interval following a 5 min seated rest (Dinamap ProCare 300 Monitor, GE Healthcare, Chicago, USA); the average of the last three measures was used. Values of blood pressure were converted into *z*-scores for age, sex, and height [19].

### 2.4. Measurement of Early Markers of CVD Development

Vascular function was assessed using Doppler ultrasound (Vivid E95, GE Healthcare, Chicago, USA) with an 11 MHz linear probe following a 10 min supine rest. Arterial stiffness was assessed with carotid-to-femoral artery PWV with the SphygmoCor (Atcor Medical, Naperville, USA) [5]. The PWV (m/s) is calculated as distance traveled by the pulse wave between the carotid and femoral arteries divided by the time it took to travel the distance [20]. A higher PWV represents higher arterial stiffness. Conversely, the lower the PWV, the more distensible the arteries. Endothelial function was estimated by brachial artery flow-mediated dilation in response to postischemic hyperemia after distal artery occlusion done by a cuff inflated at 50 mm Hg above the systolic blood pressure (SBP) measured during supine rest applied to the forearm for 5 min [21]. Indices of endothelial function included Doppler acceleration of blood flow and the velocity time integral (VTI) of the brachial artery at 15 s postcuff deflation [22]. Increased blood flow following artery occlusion suggests a greater endothelial vasodilation in response to ischemia. Thus, higher acceleration and VTI indicate better endothelial function, and conversely, lower acceleration and VTI indicate impaired endothelial function [23].

Participants underwent CMR to assess myocardial structure and function at the McGill University Health Center. CMR allows evaluation of early myocardial abnormalities in individuals living with diabetes [9]. For the quantitative assessment of cardiac morphology (including mass), volumes, and function, we applied standard cine imaging (steady-state free precession (SSFP)) in a short stack and in three long-axis views. Using a certified software (cvi42, Circle Cardiovascular Imaging Inc., Calgary, Canada), the following parameters were measured: LV end-diastolic and end-systolic volumes, LV ejection fraction, end-systolic LV mass indexed to height, papillary muscle mass, and average wall thickness of segments 1, 2, 7, and 8 [24]. Imaging evaluation was performed by two readers, with high interreader reliability based on the intraclass correlation coefficients that were all above 0.8.

### 2.5. Other Measurements

Pubertal development stage was evaluated by a trained research nurse [25, 26]. Weight (kg), height (m), and waist circumference (cm) were measured following standardized protocols [17]. Body mass index (BMI) was calculated by dividing the weight to height squared (kg/m^2^). BMI *z*-scores (zBMI) were computed based on World Health Organization reference data [27]. Overweight was defined as a zBMI ≥ 1 and <2, and obesity was defined as a zBMI ≥ 2 [27]. Percentage of body fat and the android-to-gynoid fat ratio were measured by dual-energy X-ray absorptiometry (DEXA) (Prodigy Bone Densitometer System, GE Lunar Corporation, Madison, USA). The android-to-gynoid fat ratio informs on central adiposity.

We used self-administered questionnaires in adolescents to assess tobacco consumption (never, not in the last 12 months, in the last 12 months, in the last month, every week, every day). Parental questionnaires measured mother's and father's self-identified ethnicity (White, Black African, Black American, Caribbean, Hispanic, Middle East, Asian, First Nations, other), parental history of CVD, as well as familial income and highest educational attainment of the parents (no high school diploma, high school, Cégep/vocational degree, university) as proxies for socioeconomic status.

We measured physical activity levels by 7-day accelerometry using activity monitor worn at the hip (Actigraph GT3X, Actigraph LLC, Pensacola, USA). We calculated the number of minutes per day spent in moderate-to-vigorous physical activity (MVPA) intensity using pediatric cut points [28]. The number of minutes per day spent in MVPA was averaged over the days of accelerometer wear.

### 2.6. Statistical Analyses

The CARDEA study sample size was determined to achieve a statistical power of 80% to detect an effect size of 0.5, i.e., a shift in means of 0.5 standard deviations (SD) in vascular and myocardial markers between adolescents with and without type 1 diabetes. More details on power calculations can be found in the study protocol [17].

Baseline characteristics were compared between adolescents with and without type 1 diabetes using means (SD) for normally distributed variables and medians (25th–75th percentile) otherwise. Categorical data was presented as % (*n*). CVD markers were compared between groups with Student's *t*-tests or Mann–Whitney *U* tests for the markers with a heavily skewed distribution.

We further examined the association between presence of type 1 diabetes and CVD risk factors (blood pressure and lipids) and early CVD vascular and myocardial markers in multivariable linear regression models. Triglycerides and papillary mass variables were transformed into sympercents (*y* = log_e_(*x*) × 100) because of their skewed distribution. Confounders were selected based on theoretical knowledge and included age, sex, ethnicity (White vs. other), android-to-gynoid ratio, and familial income. For PWV, endothelial function, and CMR outcomes, we additionally adjusted for systolic blood pressure *z*-score (zSBP) (all) and heart rate during the test (for PWV, acceleration, and VTI) because these outcomes are influenced by blood pressure and heart rate. To examine if the relationships between type 1 diabetes and CVD risk factors and markers differed based on sex, we tested interaction terms between type 1 diabetes status and sex in every multivariable model and estimated sex-stratified multivariable models.

Given the scarcity of knowledge on the association between type 1 diabetes status and early CVD markers, and to avoid overfitting, we opted for a parsimonious multivariable model for our main analysis. However, we performed sensitivity analyses to verify whether changes to the multivariable model would modify conclusions: (1) using percentage of body fat instead of the android-to-gynoid ratio and (2) accounting additionally for MVPA, pubertal stage (postpubertal vs. peripubertal), parental history of CVD (at least one parent with CVD vs. no parent with CVD), and tobacco consumption (smoked cigarette at least once in the last 12 months vs. not).

We performed multiple imputation to account for missing data assuming a missing at random pattern. We imputed 25 datasets using the expectation–maximization with bootstrapping algorithm in Amelia. All data were analyzed using R version 4.0.5 (R Foundation for Statistical Computing, Vienna, Austria). Statistical tests were two-sided, and significance set at the 5% level.

## 3. Results

### 3.1. Description of the Sample and Missing Data

Three hundred and fourteen adolescents with type 1 diabetes were considered eligible for study participation and of those, 100 accepted to participate and were enlisted in the CARDEA study (participation rate: 32%). A total of 97 healthy age- and sex-matched adolescents without diabetes were recruited. Characteristics of participants are presented in Table 1. Median diabetes duration was 6.1 years (25th–75th percentile 3.4–10.4) and average HbA1c was 8.5% (SD: 1.4). Adolescents with type 1 diabetes came from families with lower parental education and lower familial income and had lower MVPA levels when compared to adolescents without diabetes. Girls with type 1 diabetes had a higher zBMI, body fat percentage, and android-to-gynoid ratio than girls without diabetes. Boys with type 1 diabetes had similar zBMI than boys without diabetes, but higher body fat percentage. A greater proportion had a parental history of CVD compared to boys without diabetes. Across all variables, less than 10% of data were missing, except for PWV, where the pulse wave could not be detected in 22% of participants (Table S1).

### 3.2. Differences in CVD Risk Factors and Early CVD Markers between Groups

In univariable comparisons, adolescents with type 1 diabetes had higher diastolic blood pressure *z*-score (zDBP), as well as higher LDL-c and triglyceride levels compared with adolescents without diabetes (Table 2). Acceleration, a marker of endothelial function, was lower in adolescents with type 1 diabetes. Although the average PWV was higher in adolescents with type 1 diabetes, the difference across groups did not reach statistical significance (5.3 (SD: 0.7) vs. 5.1 (SD: 0.7) m/s, *p*-value = 0.073).

### 3.3. Associations between Type 1 Diabetes, CVD Risk Factors, and Early CVD Markers

Having type 1 diabetes was associated with a higher zDBP by 0.21 SD (95% confidence interval (CI): 0.04; 0.38), a 0.21 mmol/l higher LDL-c level (95% CI: 0.02; 0.40), a 17% higher triglyceride level (95% CI: 4; 29), and a 77.4 cm/s^2^ (95% CI: 21.6; 133.1) lower acceleration (endothelial function) when compared to adolescents without diabetes, after accounting for age, sex, ethnicity, android-to-gynoid ratio, and familial income (Table 3). Conclusions were similar when adjusting for percentage of body fat instead of the android-to-gynoid ratio and when additionally adjusting for MVPA, pubertal stage, parental history of CVD, and tobacco consumption in the models (Table S2).

### 3.4. Exploratory: Effect Modification by Sex on zSBP and Myocardial Structure

**Figure 1:**
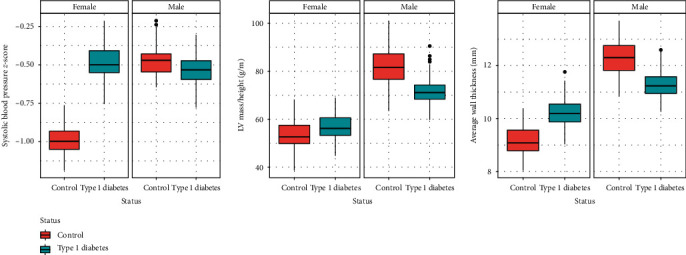
Preliminary evidence of effect modification by sex in the relationship between having type 1 diabetes and systolic blood pressure *z*-score, left ventricular mass indexed to height, and average wall thickness. LV, left ventricle. Boxplot values and effect estimates (95% CI) originate from the multivariable models with multiple imputation. We refer to Table S3 for estimated betas and 95% CI for sex-specific multivariable models with multiply imputed data.

As shown in Table 3, Figure 1, and Table S3 (for sex-specific associations), we observed that the effect of type 1 diabetes on zSBP, LV mass indexed to height, and average wall thickness was heterogeneous across sexes. Based on the sex-specific multivariable models from Table S3, having type 1 diabetes was associated with a higher zSBP (beta (95% CI): 0.35 SD (−0.03; 0.73)) in girls although the lower CI bound crossed the null value, while no association was observed in boys (beta (95% CI): −0.02 SD (−0.41; 0.37)). Girls with type 1 diabetes presented a similar LV mass indexed to height compared to girls without diabetes (beta (95% CI): 0.82 g/m (−3.37; 5.01)), while in boys, having type 1 diabetes was associated with a lower LV mass indexed to height (beta (95% CI): −8.89 g/m (−14.67; −3.11)). Having type 1 diabetes was associated with a higher average wall thickness in girls (beta (95% CI): 0.86 mm (−0.05; 1.78)), although the lower CI bound crossed the null value, and with a lower average wall thickness in boys (beta (95% CI): −1.07 (−1.92; −0.22)).

## 4. Discussion

This is the first study to our knowledge to investigate a broad spectrum of markers of both vascular and myocardial structure/function in a large number of adolescents with type 1 diabetes, using state-of-the-art measurement methods, including CMR, and to compare to adolescents without diabetes while accounting for multiple potential confounders. In addition to confirming that adolescents with type 1 diabetes have higher BP and an altered lipid profile compared to adolescents without diabetes, our study indicates that adolescents with type 1 diabetes have lower endothelial function independently of age, sex, ethnicity, central adiposity, and familial income.

Arterial stiffness by PWV was higher in adolescents with type 1 diabetes by 0.2 m/s, but differences were not statistically significant and attenuated after adjustment for confounders. In the study by Urbina et al. [4] in 535 adolescents with type 1 diabetes and 241 adolescents without diabetes, the age-adjusted mean difference of PWV was 0.3 m/s between groups. In the study by Urbina et al. [4], boys with type 1 diabetes were more likely to present abnormal peripheral and central arterial stiffness and endothelial dysfunction than girls. In contrast, we did not observe any sex-specific differences for these outcomes in our study. Dissimilar findings between Urbina's study and ours could be explained by differences in the study population and their higher sample size. Overall, our findings based on the brachial artery flow-mediated dilation test are in accordance with previous studies reporting endothelial dysfunction in children and adolescents with type 1 diabetes compared to controls based on univariable comparisons [8, 10, 29]. Consistency of our findings with previous reports, even after accounting for confounding factors (e.g., central adiposity, socioeconomic status, ethnicity), strengthens the hypothesis of the deleterious impact of type 1 diabetes on vascular function early in the course of the disease.

Effect modification by sex analyses suggested higher zSBP in female adolescents with type 1 diabetes, while no difference in zSBP across type 1 diabetes status was found in boys. In contrast, adolescent boys with type 1 diabetes seemed more likely to have elevated BP than girls based on the German diabetes surveillance program [30]. We found sex differences in myocardial structure, where boys with type 1 diabetes had a lower LV mass and a lower wall thickness compared to boys without diabetes. Differences in boys persisted even after additionally accounting for MVPA, pubertal stage, parental history of CVD, and tobacco consumption in sensitivity analyses. In contrast, girls with type 1 diabetes had a higher wall thickness than girls without diabetes, albeit not statistically significant. The higher zSBP in girls with type 1 diabetes compared to girls without diabetes could possibly explain why their LV mass was similar and wall thickness tended to be higher compared to girls without diabetes, in contrast to what we noted in boys, given the known association between higher SBP and LV enlargement. Our findings revealing differences in myocardial mass by CMR in boys with type 1 diabetes are novel and somewhat surprising. Among the few available CMR-based studies, Heier et al. [10] found no differences in LV mass, nor in markers of function (ejection fraction, filling rate), between 47 young adults with type 1 diabetes and 31 peers without diabetes. Similarly, LV mass was almost identical between 16 adolescents with type 1 diabetes and 20 adolescents without diabetes in Schäfer et al. [3].

Some of the mechanisms we can invoke for boys with type 1 diabetes having a lower LV mass when compared to boys without diabetes relate to cardiomyocyte growth and development and its association with insulin. Cardiac growth is highly regulated by insulin and the growth hormone insulin-like growth factor-1 (IGF-1) axis, which modulate nutrient availability and protein synthesis in the cell [31]. Thus, the lack of insulin in type 1 diabetes could have impaired cardiac growth in adolescents with type 1 diabetes during development. We support our hypothesis with a counterexample in the context of hyperinsulinemia. Children born to mothers with pregestational and gestational diabetes have a higher LV mass during fetal and infant life compared to children born to mothers without gestational diabetes [32]. Exposure to hyperinsulinism experienced by babies born to mothers with gestational diabetes appears to drive cardiomyocyte growth, leading to cardiac hypertrophy [32, 33]. Furthermore, adolescents with type 2 diabetes present an elevated LV mass compared to adolescents without diabetes [34]. Insulin resistance and associated compensatory hyperinsulinemia precede type 2 diabetes, such that the increased LV mass in youth with type 2 diabetes could originate from an exposure to hyperinsulinism during childhood. In contrast, the insulinopenia experienced by individuals who develop type 1 diabetes in childhood may impair glucose uptake and utilization by cardiomyocytes, and impede their growth, as reflected by the lower LV mass noted in boys with type 1 diabetes in our cohort. Finally, inherent biological differences between boys and girls that manifest at puberty onset, relating to adiposity mass and distribution and sex hormones levels, could be implicated in the myocardial development process. The interplay between these factors, type 1 diabetes, and myocardial structure needs to be further investigated in a larger sample of adolescents to better understand the role of sex in early CVD in this population.

Alternately, other mechanisms attributable to diabetes may have an impact on cardiomyocyte development. A hyperglycemic milieu favors production of advanced glycated-end products and oxidative damage [9, 35]. Adolescents with type 1 diabetes present insulin resistance [36], which causes glucotoxicity, oxidative stress, and lipotoxicity [9, 35]. Because these conditions promote cell dysfunction and apoptosis, they could alter cardiac growth during childhood and adolescence, thus leading to a lower LV mass. Evidence why these mechanisms would affect boys more than girls with type 1 diabetes remains unknown. One possible explanation relates to adiponectin, where boys with type 1 diabetes present lower levels than girls with type 1 diabetes [37]. Adiponectin lowers inflammatory responses and promotes insulin sensitivity and is associated with lower risk of CVD in adults with type 1 diabetes [38]. Overall, our findings on myocardial structure contribute to the understanding of the pathophysiological processes underlying CVD development in the pediatric population with type 1 diabetes.

Strengths of this study rely on its innovation by the assessment of early markers of vascular and myocardial structure and function in 14-18-year-olds with type 1 diabetes. This is the first study to report CMR outcomes in a large sample of adolescents with type 1 diabetes in comparison with age- and sex-matched adolescents without diabetes. Adolescents with and without type 1 diabetes were both assessed equally for vascular and myocardial structure and function, thus limiting information bias relating to measurement. In addition, we accounted for potential confounders based on theoretical knowledge in contrast to many previous studies based on CMR in pediatric type 1 diabetes with sample sizes too small to perform multivariable analyses. We used valid methods to measure many confounders, notably measuring central adiposity using the android-to-gynoid ratio by DEXA.

Some study limitations deserve mention. First, the representativeness of the group of adolescents without diabetes to the general population is limited on certain aspects. Adolescents without diabetes had parents who were more educated [39] and were less likely to be overweight or obese (21% vs. 27%) compared to the general population [40]. Thus, associations between type 1 diabetes and BP, lipid levels, and early CVD markers may be overestimated because of the comparison with healthier adolescents than those from the general population. Another limitation concerns presuppositions made about the choice of confounders and the relationships between variables. It is possible that some confounders remain unaccounted for when comparing adolescents with and without type 1 diabetes. In addition, missing data were substantial for the arterial stiffness variable PWV (22%). We performed multiple imputation to optimize the sample size and limit bias. However, we cannot discard the possibility that PWV was missing conditionally on unobserved factors or on the value of PWV itself and that imputation based on the missing at random tenant could not prevent the introduction of a bias. Last, our study was of limited power to detect small effect sizes and interactions.

## 5. Conclusions

In conclusion, in adolescents with type 1 diabetes, differences in BP, lipid levels, as well as in endothelial function and LV structure (in boys only) were already detectable at 14–18 years of age when compared to adolescents without diabetes. The role of early structural changes in the myocardium of youth with type 1 diabetes on later CVD development requires further study. Nonetheless, our findings argue for CVD prevention early in type 1 diabetes. With this aim in mind, it is essential to generate more knowledge on modifiable determinants of early vascular and myocardial alterations in adolescents with type 1 diabetes.

## Figures and Tables

**Table 1:** Characteristics of the participants in the CARDEA study.

	All			Girls			Boys		
	With type 1 diabetes	Without diabetes	*p*-Value	With type 1 diabetes	Without diabetes	*p*-Value	With type 1 diabetes	Without diabetes	*p*-Value
	(*n* = 100)	(*n* = 97)		(*n* = 48)	(*n* = 48)		(*n* = 52)	(*n* = 49)	
Age (years)	16.4 (1.3)	16.3 (1.3)	0.780	16.3 (1.4)	16.4 (1.4)	0.749	16.4 (1.5)	16.2 (1.1)	0.443
White ethnicity	62.5 (60)	75.0 (72)	0.087	63.6 (28)	78.7 (37)	0.174	61.5 (32)	71.4 (35)	0.401
Peripubertal (Tanner 1–4)	31.3 (31)	43.3 (42)	0.112	29.2 (14)	47.9 (23)	0.093	33.3 (17)	38.8 (19)	0.720
BMI *z*-score	0.75 (1.05)	0.17 (1.08)	<**0.001**	1.2 (0.9)	0.03 (0.9)	<**0.001**	0.4 (1.1)	0.3 (1.2)	0.834
Overweight or obesity	43.0 (43)	20.6 (20)	**0.001**	56.3 (27)	14.6 (7)	<**0.001**	30.8 (16)	26.5 (13)	0.802
Body fat percentage	26.5 (14.4–36.1)	21.3 (12.7–27.8)	**0.009**	36.1 (31.4–40.8)	27.7 (23.4–32.4)	<**0.001**	15.8 (11.7–21.2)	12.5 (9.1–19.3)	**0.032**
Android-to-gynoid fat ratio	0.29 (0.24–0.37)	0.27 (0.23–0.31)	**0.027**	0.32 (0.26–0.37)	0.24 (0.22–0.27)	<**0.001**	0.27 (0.22–0.35)	0.28 (0.26–0.34)	0.168
Diabetes duration (years)	6.1 (3.4–10.4)	–	–	5.2 (2.9–11.5)	–	–	6.2 (4.2–8.9)	–	–
HbA1c (%)	8.5 (1.4)	5.3 (0.2)	<**0.001**	8.6 (1.4)	5.2 (0.2)	<**0.001**	8.5 (1.4)	5.3 (0.2)	<**0.001**
Familial income^*∗*^ (USD)	40.2 K (21.7–63.5)	58.1 K (40.2–93.9)	<**0.001**	37.5 K (25.4–52.0)	55.0 K (40.2–75.0)	**0.003**	46.0 K (20.5–65.0)	77.2 K (39.6–94.2)	**0.001**
Parent(s) with university degree	52.7 (48)	80.0 (76)	<**0.001**	44.2 (19)	76.6 (36)	**0.003**	60.4 (29)	83.3 (40)	**0.023**
Parental history of CVD	41.1 (37)	26.4 (24)	0.052	29.3 (12)	28.9 (13)	1.000	51.0 (25)	23.9 (11)	**0.012**
Cigarette use in the last 12 months	18.4 (18)	15.8 (15)	0.776	19.1 (9)	12.8 (6)	0.573	17.6 (9)	18.8 (9)	1.000
Moderate-to-vigorous physical activity (min/day)	27.7 (15.3–37.8)	31.1 (24.6–48.3)	**0.001**	21.7 (11.7–32.1)	30.4 (24.4–41.9)	**0.005**	29.0 (21.0–39.7)	32.7 (24.8–52.6)	**0.048**

BMI, body mass index; CVD, cardiovascular disease; HbA1c, glycated hemoglobin. Continuous variables are presented as means (standard deviation) or medians (25th–75th percentile) if not normally distributed. Categorical variables are presented as % (*n*). Comparisons between groups were performed using Student's *t*-tests or Mann–Whitney *U* tests for continuous variables not normally distributed. Chi-square tests were used for categorical variables. Results in bold indicate *p*-value < 0.05.  ^*∗*^Familial income is divided by the square root of the number of persons living in the home.

**Table 2:** Univariable comparisons of arterial pressure, blood lipids, and early cardiovascular markers between adolescents with and without type 1 diabetes.

	All			Girls			Boys		
	With type 1 diabetes	Without diabetes	*p*-Value	With type 1 diabetes	Without diabetes	*p*-Value	With type 1 diabetes	Without diabetes	*p*-Value
	(*n* = 100)	(*n* = 97)		(*n* = 48)	(*n* = 48)		(*n* = 52)	(*n* = 49)	
Arterial pressure									
zSBP	−0.51 (0.73)	−0.73 (1.02)	0.087	−0.48 (0.66)	−0.99 (0.94)	**0.003**	−0.53 (0.79)	−0.47 (1.04)	0.746
zDBP	−0.79 (0.58)	−1.03 (0.55)	**0.004**	−0.67 (0.63)	−1.05 (0.54)	**0.002**	−0.90 (0.51)	−1.00 (0.56)	0.334
Lipids									
HDL-c (mmol/L)	1.4 (0.2)	1.4 (0.3)	0.796	1.4 (0.2)	1.5 (0.3)	0.656	1.35 (0.2)	1.30 (0.3)	0.370
LDL-c (mmol/L)	2.6 (0.8)	2.4 (0.6)	**0.024**	2.9 (0.8)	2.5 (0.6)	**0.012**	2.4 (0.6)	2.3 (0.6)	0.012
Triglycerides (mmol/L)	0.9 (0.7–1.0)	0.7 (0.6–0.9)	**0.004**	0.9 (0.7–1.2)	0.7 (0.6–0.9)	**0.001**	0.7 (0.6–1.0)	0.8 (0.6–0.9)	0.488
Arterial stiffness									
PWV (m/s)	5.3 (0.7)	5.1 (0.7)	0.073	5.3 (0.7)	5.0 (0.7)	0.165	5.3 (0.6)	5.2 (0.7)	0.298
Endothelial function									
VTI (cm)	24.5 (7.8)	27.0 (9.6)	0.053	23.6 (7.1)	26.1 (9.5)	0.173	25.3 (8.4)	27.9 (9.6)	0.157
Acceleration (cm/s^2^)	622 (169)	690 (183)	**0.008**	602 (166)	676 (175)	**0.043**	639 (172)	704 (191)	0.081
CMR									
Left ventricular ejection fraction (%)	65.9 (6.1)	64.9 (5.7)	0.217	66.0 (6.5)	64.5 (5.8)	0.234	65.9 (5.8)	65.3 (5.6)	0.602
Left ventricle mass indexed to height (g/m)	64.8 (15.3)	68.5 (19.9)	0.156	57.4 (9.2)	53.4 (9.2)	**0.042**	71.5 (16.6)	82.7 (16.6)	**0.001**
Papillary muscle mass (g)	0.35 (0.14 – 0.72)	0.50 (0.18 – 0.90)	0.089	0.29 (0.12 – 0.62)	0.58 (0.16 – 1.02)	0.076	0.39 (0.15 – 0.81)	0.47 (0.21 – 0.64)	0.497
Average wall thickness (mm)	10.8 (1.9)	10.8 (2.6)	0.927	10.2 (1.9)	9.2 (1.8)	**0.007**	11.2 (1.8)	12.4 (2.4)	**0.010**

CMR, cardiac magnetic resonance; HDL-c, high-density lipoprotein cholesterol; LDL-c, low-density lipoprotein cholesterol; PWV, pulse wave velocity; VTI, velocity time integral; zDBP, diastolic blood pressure *z*-score; zSBP, systolic blood pressure *z*-score. Continuous variables are presented with means (standard deviation) or medians (25th–75th percentile) if not normally distributed. Comparisons between groups were performed using Student's *t*-tests or Mann–Whitney *U* tests for variables not normally distributed. Results in bold indicate *p*-value < 0.05.

**Table 3:** Multivariable linear regression estimates (95% CI) on the relation between having type 1 diabetes versus not on arterial pressure, blood lipids, and early cardiovascular markers.

	Case complete analysis		Multiple imputation analysis	
	*β* coefficient for type 1 diabetes (95% CI)	*β* coefficient for type 1 diabetes by sex (95% CI)	*β* coefficient for type 1 diabetes (95% CI)	*β* coefficient for type 1 diabetes by sex (95% CI)
Arterial pressure				
zSBP^*∗*^	**0.45 (0.04; 0.86)**	−0.44 (−0.99; 0.11)	**0.40 (0.03; 0.78)**	−0.49 (−1.00; 0.03)
zDBP	**0.19 (0.01; 0.37)**	–	**0.21 (0.04; 0.38)**	–
Lipids				
HDL-c (mmol/L)	0.04 (−0.04; 0.12)	–	0.03 (−0.05; 0.10)	–
LDL-c (mmol/L)	**0.22 (0.01; 0.42)**	–	**0.21 (0.02; 0.40)**	–
Triglycerides (SYM%)	**13.6 (0.6; 26.6)**	–	**16.5 (4.2; 28.9)**	–
Arterial stiffness^†,‡^				
PWV (m/s)	0.04 (−0.18; 0.26)	–	0.05 (−0.17; 0.27)	–
Endothelial function^†,‡^				
VTI (cm)	−0.85 (−3.64; 1.93)	–	−1.84 (−4.43; 0.76)	–
Acceleration (cm/s^2^)	−**80.7 (−142.0; −19.4)**	–	−**77.4 (−133.1; −21.6)**	–
CMR^†^				
Left ventricular ejection fraction^‡^ (%)	1.28 (−0.73; 3.30)	–	0.94 (−0.91; 2.79)	–
Left ventricle mass indexed to height^*∗*^ (g/m)	−1.87 (−7.70; 3.95)	−6.59 (−14.30; 1.13)	−1.02 (−6.45; 4.40)	−**7.89 (−15.16; −0.62)**
Papillary muscle mass (SYM%)	−33.8 (−81.5; 13.9)	–	−25.6 (−68.0; 16.7)	–
Average wall thickness^*∗*^ (mm)	0.40 (−0.57; 1.37)	−**1.46 (−2.74; −0.18)**	0.50 (−0.36; 1.36)	−**1.47 (−2.65; −0.29)**

CI, confidence interval; CMR, cardiac magnetic resonance; HDL-c, high-density lipoprotein cholesterol; LDL-c, low-density lipoprotein cholesterol; PWV, pulse wave velocity; VTI, velocity time integral; zDBP, diastolic blood pressure *z*-score; zSBP, systolic blood pressure *z*-score. Models were adjusted for age, sex, ethnicity, android-to-gynoid ratio, and familial income. Triglycerides and papillary muscle mass variables were log-transformed into sympercents because of their heavily skewed distribution. Results in bold indicate *p*-value < 0.05. Beta coefficient interpretation can be interpreted as follows, using zDBP as an example, multiple imputation analysis: having type 1 diabetes is associated with a 0.21 (95% CI: 0.04; 0.38) higher zDBP compared to not having type 1 diabetes, for a fixed level of the covariates.  ^*∗*^Significant interactions terms between having type 1 diabetes and sex (males = 1, females = 0) were found for these variables. Interpretation for interaction terms goes as follow, using complete case models as an example. For zSBP: Having type 1 diabetes was associated with a 0.45 SD (95% CI: 0.04; 0.86) higher zSBP in girls, while no difference by type 1 diabetes status was observed in boys (beta (95% CI): 0.01 SD (−0.36; 0.38)). LV mass indexed to height: no difference was observed in girls (beta (95% CI): −1.87 g/m (−7.70; 3.95)). In boys, having type 1 diabetes was associated with lower LV mass indexed to height (beta (95% CI): −8.46 g/m (−13.57; −3.35)). Average wall thickness: having type 1 diabetes was associated with a higher wall thickness in girls although the lower CI bound crossed the null (beta (95% CI): 0.40 mm (−0.57; 1.37)). In boys, having a type 1 diabetes was associated with a lower wall thickness in boys (beta (95% CI): −1.06 (−1.90; −0.21)). We refer to Table S3 for estimated betas and 95% CI for sex-specific multivariable models with multiply imputed data. ^†^Additionally adjusted for systolic blood pressure *z*-score. ^‡^Additionally adjusted for heart rate during the test.

## Data Availability

The datasets generated during and/or analyzed during the current study are not publicly available due to ethics requirements but are available from the corresponding author upon reasonable request.

## Supplementary Materials

Supplementary Materials Table S1: proportion of missing data among the 197 participants. Table S2: sensitivity analyses: multivariable linear regressions on the relationship between type 1 diabetes status and arterial pressure, blood lipids, and early cardiovascular markers. Table S3: sex-specific multivariable linear regression estimates (95% CI) on the relation between having type 1 diabetes vs. not on arterial pressure, blood lipids, and early cardiovascular markers.
