# Supplementary material for: Vascular and Myocardial Structure and Function in Adolescents with Type 1 Diabetes: The CARDEA Study
Source: Pediatr Diabetes. 2023 Aug 24;2023:8662038. doi: 10.1155/2023/8662038 (PMC12017143; doi:10.1155/2023/8662038)
Supplement: Supplementary Materials — Table S1: proportion of missing data among the 197 participants. Table S2: sensitivity analyses: multivariable linear regressions on the relationship between type 1 diabetes status and arterial pressure, blood lipids, and early cardiovascular markers. Table S3: sex-specific multivariable linear regression estimates (95% CI) on the relation between having type 1 diabetes vs. not on arterial pressure, blood lipids, and early cardiovascular markers. [file 8662038.f1.docx]

**SUPPLEMENTAL MATERIAL**

Table S1. Proportion of missing data among the 197 participants

|  | Missing (%) |
| --- | --- |
| Blood pressure (zSBP, zDBP) | 0.5 |
| Serum lipids (HDL-c, LDL-c, triglycerides) | 1.5 |
| Arterial stiffness (pulse-wave velocity) | 21.8 |
| Endothelial function (VTI and acceleration) | 5.6 |
| Heart rate during brachial artery distensibility test | 2.5 |
| Left ventricular ejection fraction | 3.1 |
| Left ventricular mass indexed to height | 3.6 |
| Papillary muscle mass | 3.1 |
| Average wall thickness | 3.1 |
| Heart rate during cardiac magnetic resonance | 3.1 |
| Ethnicity | 2.5 |
| Android-to-gynoid fat ratio | 6.1 |
| Parental history of cardiovascular disease | 8.1 |
| Cigarette use in the last 12 months | 2.0 |
| Familial income | 5.1 |
| Moderate-to-vigorous physical activity (accelerometer) | 7.1 |

*Legend.* HDL-c: high density lipoprotein cholesterol, LDL-c: low density lipoprotein cholesterol, VTI: velocity time integral, zDBP: diastolic blood pressure z-score, zSBP: systolic blood pressure z-score.

Table S2. Sensitivity analyses: multivariable linear regressions on the relationship between type 1 diabetes status and arterial pressure, blood lipids, and early cardiovascular markers, case complete analysis

|  | Main analysis model | | Main model with %BF instead of android-to-gynoid ratio | | Main model + MVPA, pubertal stage, parental history of CVD, and smoking | |
| --- | --- | --- | --- | --- | --- | --- |
|  | β coef. for type 1 diabetes (95% CI) | β coef. for type 1 diabetes by sex  (95% CI) | β coef. for type 1 diabetes (95% CI) | β coef. for type 1 diabetes by sex  (95% CI) | β coef. for type 1 diabetes (95% CI) | β coef. for type 1 diabetes by sex  (95% CI) |
| Arterial pressure |  |  |  |  |  |  |
| zSBP* | **0.45 (0.04 ; 0.86)** | -0.44 (-0.99 ; 0.11) | 0.40 (-0.01 ; 0.81) | -0.45 (-0.98 ; 0.07) | **0.45 (0.01 ; 0.91)** | -0.36 (-0.99 ; 0.26) |
| zDBP | **0.19 (0.01 ; 0.37)** | - | 0.16 (-0.02 ; 0.35) | - | 0.17 (-0.03 ; 0.37) | - |
| Lipids |  |  |  |  |  |  |
| HDL-c, mmol/l | 0.04 (-0.04 ; 0.12) | - | 0.05 (-0.03 ; 0.14) | - | 0.07 (-0.02 ; 0.16) | - |
| LDL-c, mmol/l | **0.22 (0.01 ; 0.42)** | - | 0.18 (-0.04 ; 0.39) | **-** | 0.21 (-0.002 ; 0.42) | - |
| Triglycerides, SYM% | **13.6 (0.6 ; 26.6)** | - | 10.4 (-3.3 ; 24.1) | - | 13.8 (-0.2 ; 27.8) | **-** |
| Arterial stiffness^†,‡^ |  |  |  |  |  |  |
| PWV, m/s | 0.04 (-0.18 ; 0.26) | - | 0.03 (-0.20 ; 0.26) | - | 0.01 (-0.21 ; 0.24) | - |
| Endothelial function^†,‡^ |  |  |  |  |  |  |
| VTI, cm | -0.85 (-3.64 ; 1.93) | - | -1.54 (-4.38 ; 1.29) | - | -1.54 (-4.67 ; 1.58) | - |
| Acceleration, cm/s^2^ | **-80.7 (-142.0 ; -19.4)** | **-** | **-89.6 (-152.7 ; -26.5)** | **-** | **-90.1 (-157.6 ; -22.5)** | **-** |
| CMR^†^ |  |  |  |  |  |  |
| Left ventricular ejection fraction^‡^, % | 1.28 (-0.73 ; 3.30) | - | 1.05 (-1.02 ; 3.13) | - | 1.12 (-1.16 ; 3.39) | - |
| Left ventricle mass indexed to height*, g/m | -1.87 (-7.70 ; 3.95) | -6.59 (-14.30 ; 1.13) | -1.80 (-7.66 ; 4.07) | **-8.13 (-15.57 ; -0.70)** | -1.28 (-7.36 ; 4.81) | -7.73 (-15.77 ; 0.31) |
| Papillary muscle mass, SYM% | -33.8 (-81.5 ; 13.9) | - | -22.4 (-71.4 ; 26.7) | - | -41.3 (-93.1 ; 10.5) | - |
| Average wall thickness*, mm | 0.40 (-0.57 ; 1.37) | **-1.46 (-2.74 ; -0.18)** | 0.19 (-0.77 ; 1.14) | **-1.48 (-2.69 ; -0.27)** | 0.39 (-0.71 ; 1.48) | **-1.61 (-3.06 ; -0.17)** |

*Legend.* CMR: cardiac magnetic resonance, CVD: cardiovascular disease, HDL-c: high density lipoprotein cholesterol, MVPA: moderate-to-vigorous physical activity; LDL-c: low density lipoprotein cholesterol, PWV: pulse-wave velocity, VTI: velocity time integral, zDBP: diastolic blood pressure z-score, zSBP: systolic blood pressure z-score. In the first column, we show the beta coefficients and 95% CI from the model used in the main analysis, adjusting for age, sex, ethnicity, android-to-gynoid ratio, and familial income. In the second column, we verified the impact on the estimates of using percentage of body fat instead of the android-to-gynoid ratio in the model. In the third column, we verified whether accounting additionally for MVPA, pubertal stage (post-pubertal vs. peripubertal), parental history of CVD, and tobacco consumption (smoked in the last 12 months vs. not) in the model. Triglycerides and papillary muscle mass variables were log-transformed into sympercents because of their heavily skewed distribution. Results in **bold** indicate *p*-value < 0.05.

*Models shown with interaction terms between having type 1 diabetes and sex (males=1, females=0), as they were found meaningful for these variables. Interpretation for interaction terms goes as follow, using main analysis model as an example. For zSBP: Having type 1 diabetes was associated with a 0.45 SD (95% confidence interval [CI]: 0.04; 0.86) higher zSBP in girls, while no difference by type 1 diabetes status was observed in boys (beta [95% CI]: 0.01 SD [-0.36; 0.38]). LV mass indexed to height*:* no difference was observed in girls (beta [95% CI]: -1.87 g/m [-7.70; 3.95]). In boys, having type 1 diabetes was associated with lower LV mass indexed to height (beta [95% CI]: -8.46 g/m [-13.57; -3.35]). Average wall thickness: having type 1 diabetes was associated with a higher wall thickness in girls although the lower CI bound crossed the null (beta [95% CI]: 0.40 mm [-0.57; 1.37]). In boys, having a type 1 diabetes was associated with a lower wall thickness in boys (beta [95% CI]: -1.06 [-1.90; -0.21]). We refer to Supplemental Table S3 for estimated betas and 95% CI for sex-specific multivariable models with multiply imputed data.

^†^Additionally adjusted for systolic blood pressure z-score.

^‡^Additionally adjusted for heart rate during the test.

Table S3. Sex-specific multivariable linear regression estimates (95% CI) on the relation between having type 1 diabetes vs. not on arterial pressure, blood lipids, and early cardiovascular markers, multiply imputed

|  | Girls  β (95% CI) | Boys  β (95% CI) |
| --- | --- | --- |
| Arterial pressure |  |  |
| zSBP | 0.35 (-0.03 ; 0.73) | -0.02 (-0.41 ; 0.37) |
| zDBP | 0.27 (-0.01 ; 0.54) | 0.15 (-0.08 ; 0.38) |
| Lipids |  |  |
| HDL-c, mmol/l | 0.03 (-0.08 ; 0.15) | 0.05 (-0.06 ; 0.16) |
| LDL-c, mmol/l | 0.23 (-0.09 ; 0.55) | 0.10 (-0.15 ; 0.36) |
| Triglycerides, SYM% | **22.2 (2.3 ; 42.1)** | 7.6 (-9.9 ; 25.2) |
| Arterial stiffness*^,†^ |  |  |
| PWV, m/s | -0.02 (-0.46 ; 0.42) | 0.16 (-0.12 ; 0.43) |
| Endothelial function*^,†^ |  |  |
| VTI, cm | -2.67 (-6.97 ; 1.63) | -1.69 (-5.44 ; 2.06) |
| Acceleration, cm/s^2^ | -59.6 (-149.1 ; 29.8) | -66.2 (-146.4 ; 14.0) |
| CMR* |  |  |
| Left ventricular ejection fraction^†^, % | 1.46 (-1.64 ; 4.57) | 0.97 (-1.57 ; 3.50) |
| Left ventricle mass indexed to height, g/m | 0.82 (-3.37 ; 5.01) | **-8.89 (-14.67 ; -3.11)** |
| Papillary muscle mass, SYM% | -41.5 (-114.5 ; 31.4) | -38.6 (-93.3 ; 16.1) |
| Average wall thickness, mm | 0.86 (-0.05 ; 1.78) | **-1.07 (-1.92 ; -0.22)** |

*Legend.* CMR: cardiac magnetic resonance, HDL-c: high density lipoprotein cholesterol, LDL-c = low density lipoprotein cholesterol, PWV: pulse-wave velocity, VTI: velocity time integral, zDBP: diastolic blood pressure z-score, zSBP: systolic blood pressure z-score. Models were adjusted for age, ethnicity, android-to-gynoid ratio, and familial income. Triglycerides and papillary muscle mass variables were log-transformed into sympercents because of their heavily skewed distribution. Results in **bold** indicate *p*-value < 0.05.

*Additionally adjusted for systolic blood pressure z-score.

^†^Additionally adjusted for heart rate during the test.
